# Altered relationship between cortisol response to social stress and mediotemporal function during fear processing in people at clinical high risk for psychosis: a preliminary report

**DOI:** 10.1007/s00406-021-01318-z

**Published:** 2021-09-04

**Authors:** Cathy Davies, Elizabeth Appiah-Kusi, Robin Wilson, Grace Blest-Hopley, Matthijs G. Bossong, Lucia Valmaggia, Michael Brammer, Jesus Perez, Paul Allen, Robin M. Murray, Philip McGuire, Sagnik Bhattacharyya

**Affiliations:** 1 Department of Psychosis Studies, Institute of Psychiatry, Psychology and Neuroscience, King’s College London, De Crespigny Park, London, SE5 8AF UK; 2 Department of Psychiatry, University Medical Center Utrecht Brain Center, Utrecht University, Utrecht, The Netherlands; 3 Department of Psychology, Institute of Psychiatry, Psychology and Neuroscience, King’s College London, London, UK; 4 National Institute for Health Research (NIHR) Maudsley Biomedical Research Centre (BRC), South London and Maudsley NHS Foundation Trust, London, UK; 5 Department of Neuroimaging, Institute of Psychiatry, Psychology and Neuroscience, King’s College London, London, UK; 6 CAMEO Early Intervention Service, Cambridgeshire and Peterborough NHS Foundation Trust, Cambridge, UK; 7 Department of Psychology, University of Roehampton, London, UK; 8 Icahn School of Medicine, Mount Sinai Hospital, New York, USA; 9 Outreach and Support in South London (OASIS) Service, South London and Maudsley NHS Foundation Trust, London, UK

**Keywords:** Clinical high risk, Psychosis, Trier social stress test, Cortisol, Cannabidiol, HPA axis

## Abstract

Evidence suggests that people at Clinical High Risk for Psychosis (CHR) have a blunted cortisol response to stress and altered mediotemporal activation during fear processing, which may be neuroendocrine–neuronal signatures of maladaptive threat responses. However, whether these facets are associated with each other and how this relationship is affected by cannabidiol treatment is unknown. We examined the relationship between cortisol response to social stress and mediotemporal function during fear processing in healthy people and in CHR patients. In exploratory analyses, we investigated whether treatment with cannabidiol in CHR individuals could normalise any putative alterations in cortisol-mediotemporal coupling. 33 CHR patients were randomised to 600 mg cannabidiol or placebo treatment. Healthy controls (*n* = 19) did not receive any drug. Mediotemporal function was assessed using a fearful face-processing functional magnetic resonance imaging paradigm. Serum cortisol and anxiety were measured immediately following the Trier Social Stress Test. The relationship between cortisol and mediotemporal blood-oxygen-level-dependent haemodynamic response was investigated using linear regression. In healthy controls, there was a significant negative relationship between cortisol and parahippocampal activation (*p* = 0.023), such that the higher the cortisol levels induced by social stress, the lower the parahippocampal activation (greater deactivation) during fear processing. This relationship differed significantly between the control and placebo groups (*p* = 0.033), but not between the placebo and cannabidiol groups (*p* = 0.67). Our preliminary findings suggest that the parahippocampal response to fear processing may be associated with the neuroendocrine (cortisol) response to experimentally induced social stress, and that this relationship may be altered in patients at clinical high risk for psychosis.

**Supplementary Information:**

The online version contains supplementary material available at 10.1007/s00406-021-01318-z.

## Introduction

Interactions between environmental stress and brain pathophysiology are thought to drive the onset of psychosis in people at Clinical High Risk (CHR), with hypothalamic–pituitary–adrenal (HPA) axis dysfunction as a putative mediator [1, 2]. CHR individuals show increased basal cortisol levels compared to healthy controls [2, 3] (akin to tonic HPA hyperactivation [4]) but an attenuated cortisol awakening response [2, 5]. Moreover, evidence suggests a blunting of the normative cortisol response to acute stress induction (phasic HPA blunting [4]) in CHR individuals [6], which is thought to underlie their enhanced vulnerability to the deleterious effects of stress [7, 8].

The neural architecture that confers this enhanced vulnerability to the effects of (particularly social) stress is not completely clear, but dysfunction within prefrontal and mediotemporal cortex, as well as midbrain-striatal dopamine signalling, have been implicated [9]. In animals, stress (especially repeated or prolonged stress) increases the responsivity of midbrain dopamine neurons leading to striatal dopamine release [10], an effect that is normalised by inhibiting the hippocampus [11] (for reviews see [9, 12]) and which is translationally relevant given that mesolimbic hyperdopaminergia is thought to be the final common pathway to psychosis in humans [13]. Numerous psychosocial stressors associated with increased risk for psychosis [14], such as migration and childhood adversity, have been shown to augment dopamine synthesis capacity and/or release [15, 16] (although dampening has also been observed [17]), and may sensitise the mesolimbic dopamine system to future stress (for review see [18, 19]). Moreover, stress-induced striatal dopamine release is highly correlated with stress-induced salivary cortisol [20–22]. Relative to healthy individuals, CHR patients show increased striatal dopamine release in response to stress [22] which correlates with cortisol release, a relationship that appears to be decoupled in cannabis-using CHR patients [23]. Conversely, the medial prefrontal cortex and hippocampus play major regulatory roles in inhibiting stress-related mesolimbic hyperdopaminergia [9, 11, 24]. While CHR individuals appear to have normal stress-induced prefrontal dopamine release, combined with a normative correlation with stress-induced salivary cortisol [25], those CHR patients with greater stress (chronic or life events) or anxiety had both lower prefrontal dopamine release as well as blunted cortisol responses following stress [25].

However, accumulating evidence suggests that it is hippocampal dysfunction that drives the downstream pathophysiology and hyper-responsivity of the dopamine system in psychosis and CHR states [26, 27]. Repeated stress exposure, a known risk factor for psychosis onset, impacts the function and structural integrity of hippocampus [28], which is rich in glucocorticoid receptors [29] and plays a fundamental role in HPA axis regulation [30]. Mediotemporal dysfunction is also strongly implicated in established models of psychosis pathogenesis [31, 32]. In healthy people, stress-induced cortisol release has been associated with deactivation of limbic structures during fear- and stress-related functional magnetic resonance imaging (fMRI) tasks (for review see [33]), and this coupling has been observed when the stress task is conducted simultaneously inside the scanner environment [34, 35] as well as when fMRI and stress induction are performed separately [36]. Models suggest that mediotemporal deactivation is a necessary requirement for the HPA axis response to stress, and that failure to deactivate the hippocampal formation in particular (i.e., hippocampus proper and parahippocampal gyri) may be a neural signature of maladaptive regulatory response [34, 35, 37]. Failure to regain neural homeostasis upon challenge (allostasis) [38] may lead to enhanced vulnerability to the negative effects of stress, due to prolonged exposure and less adaptative behavioural/psychological responses. In CHR individuals, this increased sensitivity and/or lack of resilience to the effects of stress may then worsen existing pathophysiology and contribute to the onset of psychosis [2, 39].

In our previous report, we found that relative to healthy controls, CHR individuals under placebo conditions had a blunted cortisol response and exaggerated anxiety response to the Trier Social Stress Test (TSST) [40]. Moreover, these neuroendocrine and psychological alterations could be partially attenuated by a 7-day course of cannabidiol (CBD)—a non-intoxicating constituent of the cannabis plant with anxiolytic [41, 42] and antipsychotic properties [43–47]. We also recently reported that CHR individuals show altered neural response to fear processing compared to healthy controls in mediotemporal limbic structures and the striatum [48]—regions strongly implicated in psychosis pathogenesis [31, 32]. In addition, we found that a single dose of CBD (in a parallel group of CHR individuals) attenuated the deviation in neural responses in the same brain regions [48]. While this and previous work points to (a) alterations in neuroendocrine and psychological response to stress and (b) altered neural response during fear processing in CHR patients, whether (and how) these facets are associated with *each other* has not been tested before. In addition, whether CBD affects the putative alterations in cortisol-mediotemporal coupling in CHR individuals remains to be evaluated.

Conceptually, fear and stress responses are distinct—but closely intertwined—biological processes that overlap and interact on a neuronal as well as neuroendocrine level [49]. On one hand, the HPA axis stress response includes a range of behavioural and physiological phenomena (such as cortisol/stress-hormone secretion, autonomic arousal) in response to threatening/arousing stimuli or homeostatic challenge [50]. Fear processing, on the other hand, involves the perception, assessment, learning and execution of appropriate responses to cues that signal danger and imminent threat (or even potential/perceived threat, in the case of fearful-face viewing) [51, 52], which may or may not provoke an HPA-axis response depending on stimulus attributes and contextual factors [53]. On a neuronal level, the neural substrates of fear overlap with the neurocircuitry that orchestrates the stress response (for review see [49]). For example, outside of the hypothalamus, the hippocampus, amygdala and prefrontal cortex are major regulatory nodes of the HPA axis as well as core components of fear-processing circuitry [30]. Fear and stress also appear to interact on a neuroendocrine level. Once the HPA axis is activated, its terminal product, cortisol, is released and binds receptors throughout the limbic system, including hippocampus, amygdala and prefrontal cortex, where it facilitates negative feedback [30, 37, 53, 54]. Fear can activate the HPA-axis and thus cortisol release, while cortisol administration has been shown to modulate fear processing, including return of fear following extinction [55–57]—potentially via effects on (para)hippocampal-amygdala function [55, 56]—as well enhancement of extinction-based psychotherapy [58]. However, fear and stress responses do not have to co-occur: a cortisol response can occur without fear, and fear can be experienced without a cortisol response [49, 59].

On the basis of previous literature, we predicted that the neural (mediotemporal) response to fear processing as indexed using fMRI would be coupled with the neuroendocrine (cortisol) and symptomatic (anxiety) response to experimentally induced social stress, and that these relationships would be altered in CHR patients. In further exploratory analyses for future hypothesis generation, we also examined whether treatment with CBD in a separate group of CHR patients would affect (i.e., ‘normalise’) any CHR-related deviation in the aforementioned relationships.

## Patients and methods

### Participants

The study received Research Ethics approval and all participants provided written informed consent. Thirty-three antipsychotic-naive CHR individuals, aged 18–35, were recruited from specialist early detection services in the United Kingdom. CHR status was determined using the Comprehensive Assessment of At-Risk Mental States (CAARMS) criteria [60]. Nineteen age (within 3 years), sex and ethnicity-matched healthy controls were recruited locally by advertisement. Exclusion criteria included history of psychotic or manic episode, current DSM-IV diagnosis of substance dependence (except cannabis), IQ < 70, neurological disorder or severe intercurrent illness, and any contraindication to MRI or treatment with CBD. Inclusion/exclusion criteria were pre-specified. Participants were required to abstain from cannabis for 96 h, other recreational substances for 2 weeks, alcohol for 24 h and caffeine and nicotine for 6 h before attending. A urine sample prior to scanning was used to screen for illicit drug use and pregnancy.

### Design, materials, procedure

CHR participants were enrolled in a randomised, double-blind, placebo-controlled, parallel-arm study [61]. Sixteen CHR participants were randomised to 600 mg oral CBD daily and 17 to identical placebo capsules (THC-Pharm). On the first day of the study, psychopathology was measured at baseline (before any drug administration) using the CAARMS (positive and negative symptoms) and State-Trait Anxiety Inventory (STAI) State Subscale [62]. Following a standard light breakfast, participants were administered the first capsule (at ~ 11 AM) and 180 min later, underwent fMRI while performing a fearful-faces task (below). Plasma CBD levels were sampled at baseline (before taking the study drug) and at 120 and 300 min after drug administration. The CHR groups then received daily CBD or placebo for 7 days. MRI data were collected in healthy controls under identical conditions but they did not receive any drug.

**Fig. 1:**
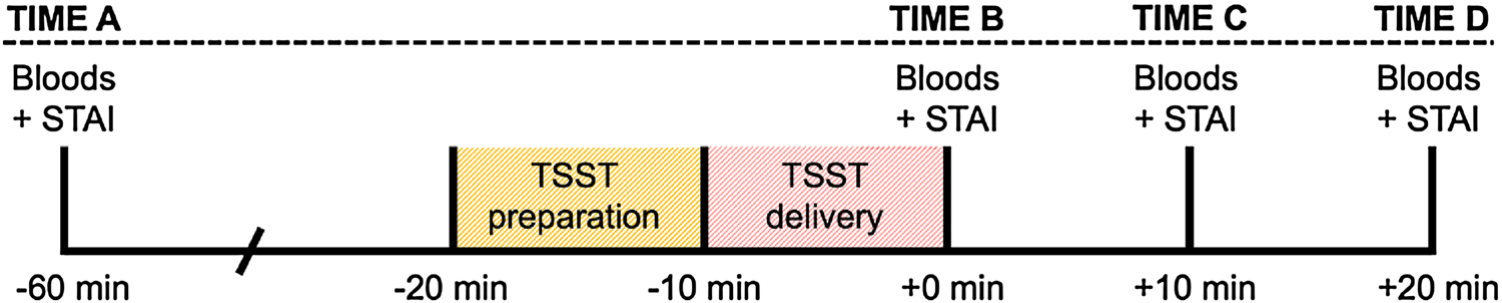
Timing of Trier Social Stress Test (TSST) procedures

On the eighth day of the study, CHR participants took part in the Trier Social Stress Test (TSST; below). Healthy participants came in for one study session only (including TSST and MRI) and were not in any drug trial. After eating breakfast at approximately 8.30 am, all participants started the TSST-day protocol (see Fig. 1) at approximately 10 am (− 60 min to TSST). As illustrated in Fig. 1, neuroendocrine response to stress was indexed by measuring serum cortisol level in blood samples collected at four time points: − 60 min (time A; baseline) and at + 0 (time B), + 10 (time C) and + 20 min (time D) after the TSST. The STAI-state was collected at each of the same time points. Specifically, participants were seated in a phlebotomy chair and a cannula was inserted into the antecubital region of the non-dominant arm. Baseline (− 60 min; time A) blood samples (2 ml) were collected into serum-separating tubes and the STAI was completed. At approximately 11 am (− 20 min), the participant took part in the TSST. Participants were led back to the phlebotomy chair and blood samples were obtained immediately (+ 0 min; time B) and the STAI was completed. Blood samples and the STAI were also completed at + 10 (time C) and + 20 min (time D). At the end of the session, participants were debriefed and received reimbursement for their participation.

### Trier social stress test

The TSST [63] is a well-validated stress induction paradigm that has been shown to reliably induce stress as reflected by changes in cortisol levels under experimental conditions (for full TSST protocol see Supplementary Material). In brief, participants were taken in front of a panel of two people and told, via standardised instructions, that they will take part in a public speaking exercise in front of the panel, with 10 min to prepare. Once they had delivered the speech, they were informed that they would take part in a mental arithmetic task as per the TSST protocol.

### Functional magnetic resonance imaging

Full details of image acquisition, fMRI task, preprocessing and fMRI analyses are detailed in the Supplementary Material and in our related publication [48]. In brief, participants were studied in one 6-min fMRI experiment at 3 T while performing a fearful face processing task. The blood-oxygen-level-dependent (BOLD) haemodynamic response was measured while subjects viewed fearful faces (mild fear, intense fear), which were contrasted with faces with neutral expressions. fMRI data were analysed with XBAM 4.1 software, using a nonparametric (permutation-testing) approach to minimise assumptions. Group (control, CBD, placebo) activation maps for fearful vs neutral conditions were compared using nonparametric analysis of variance (ANOVA) [64] to examine linear relationships in brain activation (placebo group > CBD group > control group; or placebo group < CBD group < control group). A region-of-interest (ROI) approach was used after constructing a single ROI mask of limbic structures within bilateral medial temporal cortex (hippocampus, parahippocampal gyrus and amygdala) and striatum, with striatal findings omitted from the present study. These regions were selected a priori based on our previous findings [48, 61]. The voxel-wise statistical threshold was set at *p* = 0.05, and the cluster-wise thresholds were adjusted to ensure that the number of false-positive clusters per brain would be less than 1; clusters that survived this critical statistical threshold and the corresponding *p* values are reported. Statistical (SSQ) values (see Supplementary Material) were extracted from significant limbic clusters within the mediotemporal ROI network and used in subsequent regression analyses with the cortisol and anxiety data (below). For completeness, corresponding results from wholebrain analyses are appended in the Supplementary Material.

### Statistical analyses

Our primary aim was to determine (a) how closely the mediotemporal response to fear processing is coupled with the neuroendocrine (cortisol; primary outcome) and symptomatic (anxiety; secondary outcome) response to social stress in healthy controls, and (b) whether CHR individuals show alterations in this coupling. A further, exploratory aim, with a view to generating future hypotheses, was to examine whether any absence or deviation of such coupling relationships in CHR individuals could be partially ‘restored’ by treatment with CBD.

Normative relationships between TSST-induced cortisol/anxiety levels (at time B) and mediotemporal activation during fear processing (SSQ values) were examined using linear regression in healthy controls. Time B data, which occurred immediately following the TSST (+ 0 min, but 20 min since subjects were described the components of the public speaking task in detail) was used to index the anticipatory and immediate reactive response following stress exposure, based on meta-analytic findings that cortisol levels peak 0–20 min following stressor onset in healthy individuals [65]. Group differences in the relationship between TSST-induced cortisol/anxiety (dependent variables) and mediotemporal response (SSQ values; predictors) were examined using (group-by-SSQ) interaction terms in linear regression analyses. Regressions were conducted separately for cortisol and anxiety outcomes. In the case of missing cortisol data, last observation carried forward was used to impute missing values. Analyses were conducted using RStudio 1.3.1 and statistical significance was set at *p* < 0.05 (two-tailed). We did not correct for multiple comparisons as this study is exploratory in nature and has a limited sample size; our findings and estimates should therefore be interpreted as hypotheses-generating rather than confirmatory. Details of supplementary analyses are provided in the Supplementary Material.

## Results

Some of the data reported herein have been used previously (to address different hypotheses) in our related publications [40, 48] but whereas the two CHR groups are the same as those we have previously reported on, the healthy control group is only partially overlapping. In the present study, there were no between-group differences in the majority of demographic and baseline clinical characteristics, except for fewer years of education in the placebo group relative to controls (Table 1). In the CBD group, mean plasma CBD levels were 126.4 nM (SD = 221.8) and 823.0 nM (SD = 881.5) at 120 and 300 min after drug intake, respectively. Three CHR individuals exited the scanner prior to the fMRI task and 1 did not complete the TSST, leaving 15 subjects in the placebo group and 14 in the CBD group with both TSST and fMRI data. Three healthy controls either did not complete the TSST or had insufficient cortisol/STAI data to be included in any analyses, leaving 16 healthy controls.

**Table 1:** Sociodemographic and Clinical Characteristics at Baseline

Characteristic	CBD (*n* = 16)	PLB (*n* = 17)	HC (*n* = 19)	Pairwise comparison		
				HC vs PLB	PLB vs CBD	HC vs CBD
Age, years; mean (SD)	22.7 (5.08)	24.1 (4.48)	24.3 (4.73)	*p* = 0.87^a^	*p* = 0.42^a^	*p* = 0.33^a^
Sex, *N* (%) male	10 (62.5)	7 (41.2)	10 (52.6)	*p* = 0.49^b^	*p* = 0.22^b^	*p* = 0.56^b^
Ethnicity, *N* (%)						
White	10 (62.5)	7 (41.2)	7 (36.8)	*p* = 0.77^b^	*p* = 0.43^b^	*p* = 0.25^b^
Black	2 (12.5)	5 (29.4)	4 (21.1)			
Asian	0 (0)	1 (5.9)	3 (15.8)			
Mixed	4 (25)	4 (23.5)	5 (26.3)			
Education, years; mean (SD)^c^	14.4 (2.71)	12.6 (2.76)	16.3 (2.47)	***p* < 0.001**^a^	*p* = 0.06^a^	*p* = 0.05^a^
CAARMS score, mean (SD)						
Positive symptoms	40.19 (20.80)	42.94 (29.47)	NA	NA	*p* = 0.76^a^	NA
Negative symptoms	23.25 (16.49)	28.41 (20.49)	NA	NA	*p* = 0.43^a^	NA
STAI-S, mean (SD)	40.31 (9.07)	38.94 (10.18)	NA	NA	*p* = 0.69^a^	NA
Urine drug screen results, *N* (%)						
Clean	10 (63)	8 (47)	13 (68)	*p* = 0.28^b^	*p* = 0.45^b^	*p* = 0.73^b^
THC	2 (13)	5 (29)	2 (11)			
Morphine	1 (6)	0 (0)	0 (0)			
Benzodiazepines	0 (0)	1 (6)	0 (0)			
PCP	0 (0)	1 (6)	0 (0)			
Missing	3 (19)	2 (12)	4 (21)			
Current nicotine use, *N* (%) yes	9 (56.3)	5 (29.4)	7 (36.8)	*p* = 0.64^b^	*p* = 0.12^b^	*p* = 0.25^b^
Current cannabis use, *N* (%) yes	7 (43.8)	7 (41.2)	4 (21.1)	*p* = 0.19^b^	*p* = 0.88^b^	*p* = 0.15^b^
Handedness, *N* (%) right	14 (87.5)	17 (100)	19 (100)	NA^d^	*p* = 0.16^b^	*p* = 0.11^b^

Significant differences are indicated in bold

*CAARMS* Comprehensive assessment of at-risk mental states, *CBD* cannabidiol, *PLB* placebo group, *HC* healthy control group, *N* number of subjects, *NA* not applicable, *PCP* phencyclidine, *STAI-S* State-Trait Anxiety Inventory-State Subscale (day 1, pre-drug), *THC* Δ9-tetrahydrocannabinol

^a^Independent *t* test

^b^Pearson chi-squared test

^c^Data for 1 CHR-placebo and 3 controls were missing

^d^No statistics necessary

### fMRI results

**Fig. 2:**
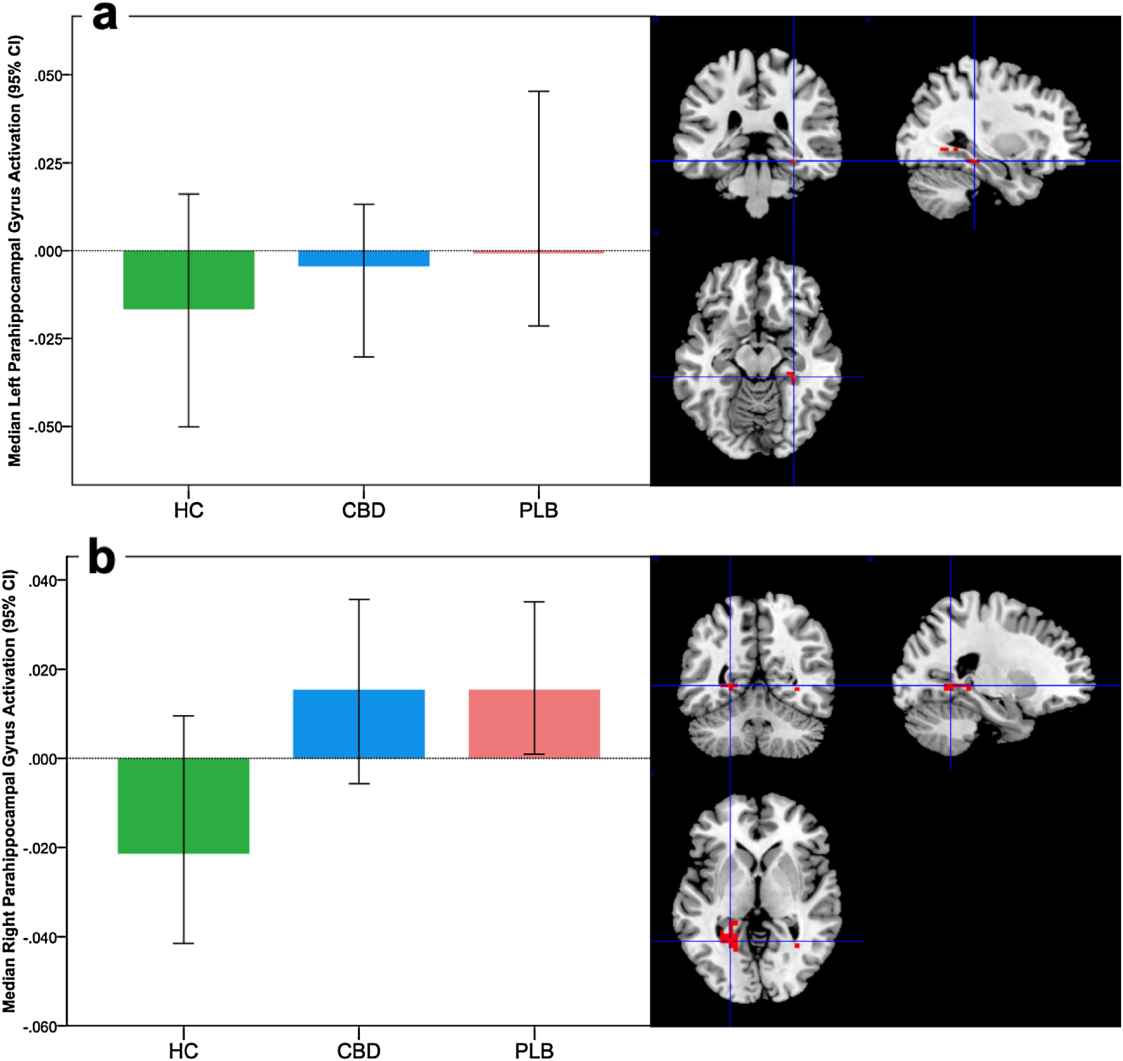
Brain activation during fear processing in CHR participants and healthy controls and the effect of cannabidiol. Clusters where activation differed across the three groups (*HC* controls, *PLB* placebo, *CBD* cannabidiol) in a linear relationship during fear processing. Median activation in each group in **a** the left parahippocampal gyrus, and **b** the right parahippocampal gyrus during fear processing, in arbitrary units as indexed using the median sum of squares ratio. The sum of squares ratio statistic refers to the ratio of the sum of squares of deviations from the mean image intensity due to the model (over the whole time series) to the sum of squares of deviations due to the residuals. The right side of the brain is shown on the left of the images

While the fMRI results themselves were not the primary aim of this study, for completeness and comparison (and to show general agreement) with our related paper [48], we report them briefly here (and in Supplementary Results). We found three significant medial temporal lobe clusters. A left parahippocampal gyrus cluster (peak Talairach coordinate *X* = − 22, *Y* = − 26, *Z* = − 17; *k* = 6; *p* < 0.001) demonstrated a linear pattern of activation across the three groups, such that healthy controls showed the greatest deactivation of this region in response to fear processing, CHR participants receiving placebo had the least deactivation, and CHR participants in the CBD group showed an intermediate level of (de)activation (Fig. 2a). In a second cluster, in the right parahippocampal gyrus (peak Talairach coordinate *X* = 25, *Y* = − 48, *Z* = − 3; *k* = 26; *p* < 0.001), healthy controls showed deactivation in response to fear processing, whereas both CHR groups showed augmented activation, with the CBD group falling marginally (but significantly) intermediate between the healthy controls and CHR placebo group (Fig. 2b). A third cluster, with a peak in the fusiform gyrus, was omitted from regression analyses as it was not within our core limbic regions of interest.

### Cortisol and fMRI response

Cortisol data were imputed for three subjects in the CBD group and two in the placebo group (Table S3 in Supplementary Material). In healthy controls, there was a significant relationship between immediate post-TSST cortisol levels and right parahippocampal activation during fear processing (B = − 4179.50, SE = 1615.46, *t* = − 2.59, *p* = 0.023), such that the greater the acute neural response (right parahippocampal deactivation), the higher the cortisol levels immediately following experimental social stress. No such relationship was observed for the left parahippocampal gyral cluster (B = − 167.63, SE = 1279.53, *t* = − 0.13, *p* = 0.90). All subsequent analyses therefore included only the right parahippocampal gyrus.

**Fig. 3:**
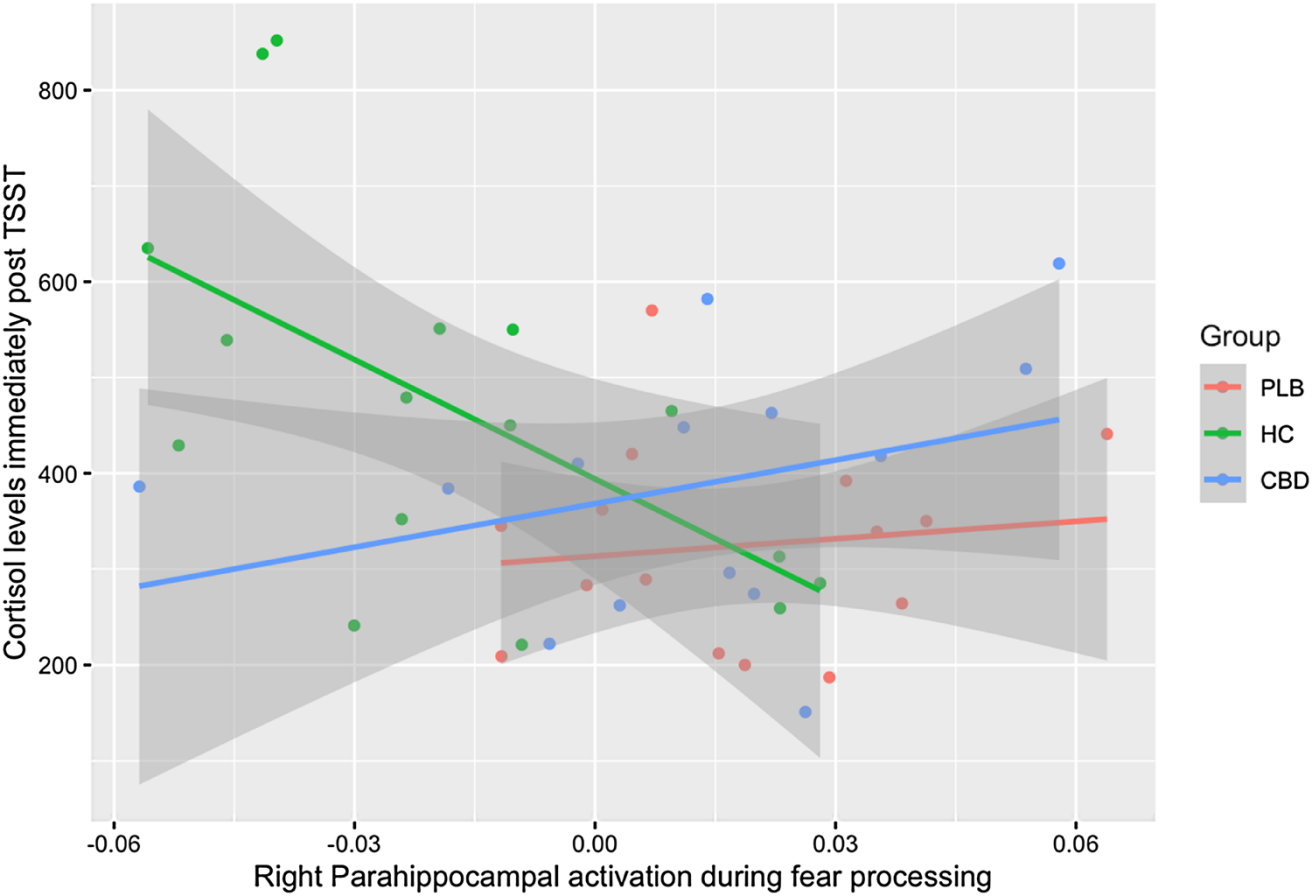
Relationship between TSST-induced cortisol and fear-related right parahippocampal activation. Relationship between TSST-induced cortisol and fear-related parahippocampal activation differed significantly between the healthy control (HC) and placebo (PLB) groups (interaction *p* = 0.033), but not between the PLB and cannabidiol (CBD) groups

In the regression model including all groups, there was a significant interaction between group (control vs placebo) and right parahippocampal activation on cortisol levels (*p* = 0.033; Table 2; Fig. 3), indicating that the relationship between cortisol and parahippocampal response was significantly different between these groups. Conversely, the relationship did not differ between the CBD and placebo groups (*p* = 0.67). For completeness, the mean cortisol response for each group over the course of the TSST, as well as supplemental analyses, are provided in the Supplementary Material.

**Table 2:** Regression model showing the effect of group, right parahippocampal activation and their interaction on TSST-induced cortisol levels

Predictor	Estimate	Std. error	*t* value	*p* value
Group (HC)	80.53	62.28	1.293	0.2036
Group (CBD)	54.82	61.68	0.889	0.3795
Parahippocampal SSQ	604.98	1702.03	0.355	0.7242
Group (HC): parahippocampal SSQ	− 4759.54	2146.70	− 2.217	**0.0325**
Group (CBD): parahippocampal SSQ	911.90	2146.33	0.425	0.6733

Results of linear regression analysis examining group differences in the relationship between cortisol levels immediately following the TSST and right parahippocampal activation (SSQ values), with the placebo group as the reference group. Significant effects are indicated in bold. *p* values are uncorrected for multiple comparisons

### Anxiety and fMRI response

**Fig. 4:**
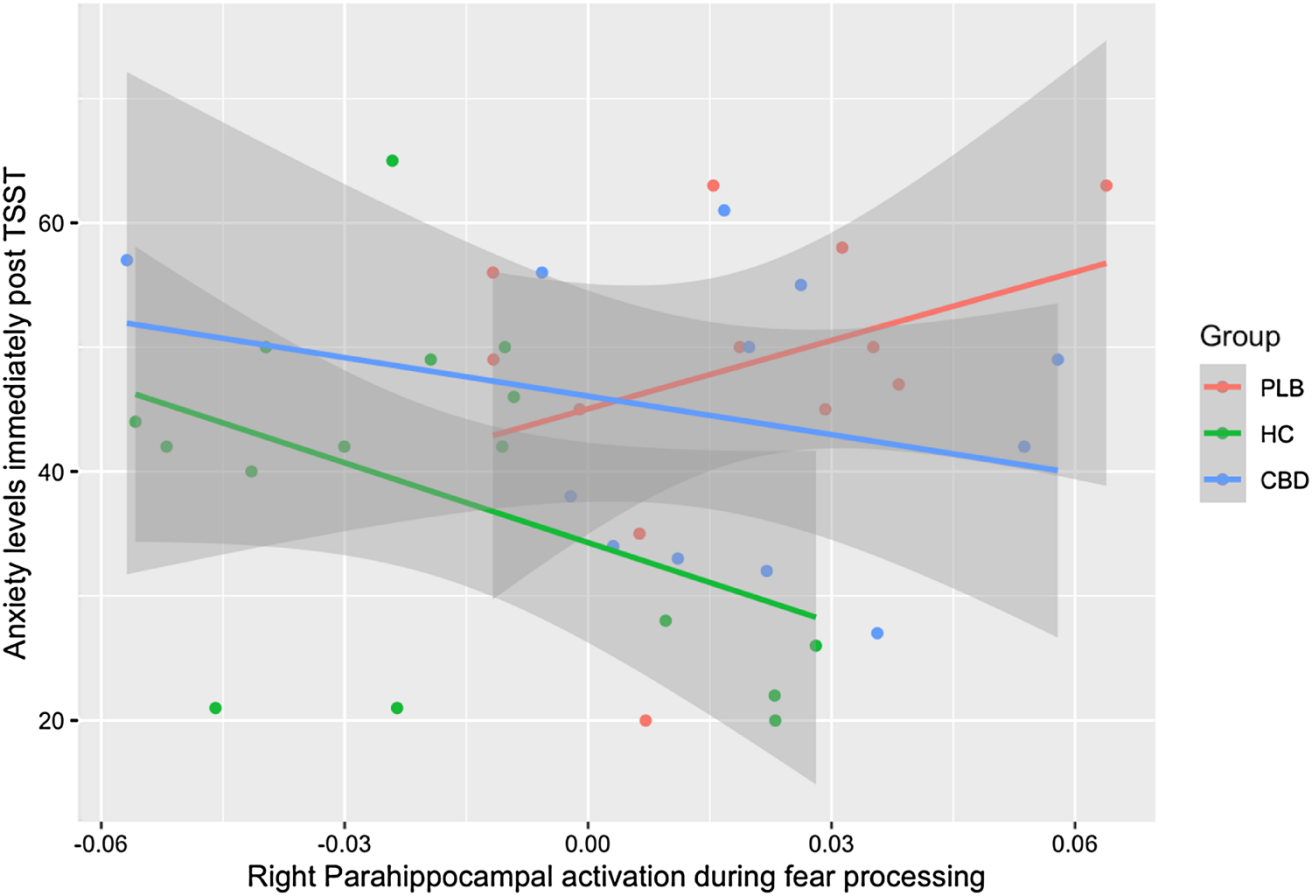
Relationship between TSST-induced anxiety and fear-related parahippocampal activation in the placebo (PLB), cannabidiol (CBD) and healthy control (HC) groups

Three placebo subjects and two CBD subjects had missing STAI data (Table S4 in Supplementary Material). In healthy controls, the relationship between TSST-induced anxiety (STAI-state scores) and right parahippocampal activation during fear processing was not significant (B = − 213.92, SE = 119.00, *t* = − 1.80, *p* = 0.094; simple linear regression). In the regression model including all groups, the relationship between right parahippocampal activation and TSST-induced anxiety did not differ significantly between the control vs placebo group (*p* = 0.054) nor between the placebo vs CBD group (*p* = 0.17) (Table 3; Fig. 4). For completeness, mean group anxiety scores across the course of the TSST, as well as supplemental analyses, are provided in the Supplementary Material.

**Table 3:** Regression model showing the effect of group, right parahippocampal response and their interaction on TSST-induced anxiety (STAI-state scores)

Predictor	Estimate	Std. error	*t* value	*p* value
Group (HC)	− 10.761	5.855	− 1.838	0.0748
Group (CBD)	1.021	6.055	0.169	0.8671
Parahippocampal SSQ	183.410	162.912	1.126	0.2681
Group (HC): parahippocampal SSQ	− 397.328	199.429	− 1.992	0.0544
Group (CBD): parahippocampal SSQ	− 286.649	202.798	− 1.413	0.1666

Results of linear regression analysis examining group differences in the relationship between anxiety (STAI-state scores) immediately following the TSST and right parahippocampal activation (SSQ values), with the placebo group as the reference group. *p* values are uncorrected for multiple comparisons

## Discussion

Our key—albeit preliminary—findings are that in healthy individuals, there exists a coupling between the mediotemporal response during fear processing and the neuroendocrine (cortisol) response to experimentally induced social stress, and that this relationship may be altered in patients at clinical high risk for psychosis (CHR). Specifically, in healthy controls, we found that the parahippocampal gyrus deactivates during fear processing and the degree of deactivation was associated with levels of cortisol released in response to social stress. In contrast, CHR individuals under placebo conditions failed to deactivate the parahippocampal gyrus in response to fear (or showed attenuated deactivation) and the relationship with stress-induced cortisol was absent.

Our finding of cortisol-neuronal coupling is in line with the majority of studies that have assessed the relation between mediotemporal function and stress-induced cortisol levels [37]. A recent systematic review concluded that exogenous cortisol administration is associated with changes in hippocampal activation during various (including emotion/fear-processing) fMRI tasks [33]. Moreover, higher levels of endogenous cortisol and greater stress-induced cortisol release are associated with hippocampal and amygdala function during/following psychological stress, with some studies finding an increase in the BOLD response and others a decrease [33].

Akin to the present findings, using both PET and fMRI Pruessner and colleagues observed profound deactivation of the limbic system (including hippocampus) in healthy individuals during an acute psychosocial stress task, with the degree of hippocampal deactivation correlating with the amount of cortisol released in response to the task [34]. These findings were later replicated [35] and commensurate results were recently found in a large sample of adolescents (*n* = 101) [66]. Similar patterns have also emerged in other niches of social stress research [67]. Based on this and other evidence, a model has been proposed in which the hippocampus is active during the default resting state, continuously evaluating sensory stimuli for signs of threat or danger [35, 68, 69]. Upon detection of salient threat or stressor onset, hippocampal deactivation is triggered as part of a core response, diverting hippocampal metabolic resources to adaptive task-specific brain regions and leading to disinhibition (i.e., initiation) of the HPA axis cortisol response [34, 35]. Preclinical evidence supports this idea and is consistent with an established function of the hippocampus—in animals and man—as a critical regulator/inhibitor of the HPA axis [30]. Activation of limbic regions has also been associated with other markers of HPA axis function. In healthy individuals, those with a greater amplitude of diurnal cortisol, indicative of healthy/normative function, show less activation in the hippocampus and amygdala during negative emotion stress-related images [70]. Fear conditioning paradigms provide complementary evidence: during early fear extinction learning, administration of cortisol has been associated with reduced activation in the amygdala–hippocampal complex and enhanced functional connectivity between the parahippocampal gyrus and prefrontal cortex [55]. Experimental stress as well as cortisol administration have also been found to attenuate amygdala–hippocampal complex activation in males during fear acquisition and reward anticipation [71–73]. However, despite our findings of mediotemporal *deactivation* in response to fear, numerous studies provide evidence of *increased* activation to fear and stress-related conditions, sometimes correlating positively with cortisol release. One study found that procaine hydrochloride—a pharmacological probe that induces powerful emotional, autonomic and endocrine responses—selectively *activated* the anterior limbic and paralimbic network, increased serum cortisol and induced subjective fear, anxiety and panic [74]. Direct electrode stimulation of mediotemporal structures in patients with epilepsy shows that fear is the most common phenomena elicited by amygdala and parahippocampal activation [75–77]. Using more similar methods to the current analyses, one study found that greater cortisol response to stress was associated with *greater* mediotemporal activation during emotional face processing [78]. Greater cortisol reactivity to fear-related emotional imagery has also been associated with greater BOLD signal in the amygdala [79, 80] and hippocampus and lower activation in the prefrontal cortex [79]. Many other studies have found no association between cortisol and mediotemporal BOLD signal during psychosocial stress tasks [81–83] (for review see [33]). Finally, while the current study focused on mediotemporal structures, it is important to note that studies and neuroimaging meta-analysis have implicated further regions in stress responses, such as the inferior frontal gyrus, insula and striatum [84], and endogenous cortisol has been associated with further regions including the anterior cingulate and inferior temporal gyrus [33]. Nevertheless, and irrespective of the directionality, which may be influenced by many task, sample and methodological factors, the present findings add to this literature by showing that cortisol response to social stress may be related to brain response to fear processing, even when measured in separate experimental paradigms.

It is worth noting that within studies of healthy people, not all individuals respond in the same way [85]; hippocampal deactivations and correlations with cortisol release have sometimes been observed only in ‘stress-responsive’ individuals, i.e., those whose cortisol increases following a stressor [34]. In healthy people, the factors and mechanisms underlying interindividual differences in stress responsivity are likely numerous [78, 86], and it is not clear whether they overlap with—or are distinct from—the factors underlying dysregulated stress responsivity in people at CHR. The normative cortisol response to acute stress is that of a peak following a stressor and a gradual diminution thereafter [86]. It could well be that in healthy individuals, attenuated or relatively non-exaggerated stress responses (e.g., relatively lower amplitude of TSST-induced cortisol release) are adaptive, in that external challenges to homeostasis are dealt with swiftly and using the minimally sufficient resources, thereby avoiding prolonged exposure to the effects of corticosteroids [86]. For example, one study found that better psychological resources and lower TSST-induced cortisol levels were associated with dampening of amygdala response to emotional faces in healthy individuals [36]. Conversely, the mechanism(s) underlying altered stress responsivity in patients at CHR may be pathological in nature rather than efficient, arising through dysfunction or desensitisation of the HPA axis, with an insufficient stress response causing prolonged exposure to the negative effects of stress and less effective behavioural/psychological responses [2, 6]. This notion is supported by findings that cortisol levels during the TSST are significantly lower in CHR patients compared to controls, with lower cortisol levels associated with higher self-ratings of stress in the past year, and chronic stress in the past month associated with higher levels of psychiatric symptoms, depression and lower self-esteem [6]. In the present study, supplementary analyses revealed significantly greater anxiety (measured using area under the curve using all four timepoints, see Supplementary Material), against a backdrop of significantly blunted cortisol response in the CHR-placebo group compared to controls. Moreover, in our previous publication using the same CHR sample, the blunted cortisol response to the TSST in CHR individuals was also associated with greater psychological response in terms of anxiety and perception of public speaking as more stressful [40]. Together, these findings are consistent with the notion that blunted TSST-induced cortisol responses in CHR individuals are maladaptive and related to worse psychological sequalae following stress.

The idea that limbic deactivation is perhaps essential for initiation or proper functioning of the cortisol response to stress is interesting in light of the differences we observed between healthy individuals and those at CHR. In our previous but related paper (detailing the main effects of the fMRI task used in the present study), we showed that fear processing was associated with deactivation of the parahippocampal gyrus in healthy controls [48]. We then demonstrated that CHR individuals have a significantly different neural response to fear, showing increased activation (rather than deactivation) in bilateral parahippocampal gyri [48]. Together, the preliminary findings of the present study extend our previous results to suggest that the normative relationship between stress-induced cortisol and the neural response to fear processing may be altered in CHR individuals.

Few, if any, previous studies have directly examined altered neural responses and stress-induced cortisol function in CHR individuals, but various disparate strands of evidence support the view that they may be related. In structural MRI studies of CHR individuals, reduced post-awakening cortisol response is associated with smaller grey matter volumes in the parahippocampal gyri [87] and hippocampus [88]. In siblings of patients with psychosis, smaller hippocampal volumes are associated with increased emotional and cortisol responses to daily life stress [89]. In functional MRI studies, first-degree siblings showed abnormal brain activation during an emotional processing fMRI task which occurred 30 min following the TSST; whereas healthy controls robustly deactivated core salience and default mode network regions, there was no such deactivation in siblings [90]. The ability to dynamically shift away from default mode network function (where the hippocampal region is a key node) following stress has been proposed as a neural signature of adaptive recovery, with the absence of this neuronal resource reallocation potentially increasing vulnerability to stress, either through direct physiological or psychological (i.e., increased rumination) mechanisms [90]. In the present study, the failure of CHR individuals to deactivate the parahippocampal gyri is in keeping with established models which propose that mediotemporal hyperactivation is critical to psychosis onset [31, 32, 91–93], and is consistent with previous evidence of elevated limbic response in those with psychosis-spectrum features [94] and individuals at genetic risk [95].

The precise mechanisms underlying the lack of coupling between neural response to fear and cortisol response to stress in CHR individuals are unclear. In line with previous findings, it is possible that both the failure to deactivate parahippocampal regions during fear processing and the failure to mount an adaptive cortisol response to social stress share common underlying substrates, or arise through similar generic deficits in responding optimally to arousing/stressful stimuli. On the other hand, it is also possible, although we think less likely, given the patterns observed in previous literature, that the correlation in healthy controls (that is absent in CHR individuals) may be driven by unrelated (third variable) factors. However, the present exploratory study was not designed to examine this, which may be a focus in future research. Relatedly, it is possible that the lack of correlation in CHR groups is due to range restriction in the cortisol and/or parahippocampal activation values in the CHR groups. While we cannot be completely sure that this is not the case, examination of the data suggests that the parahippocampal activation values are not range restricted and variability does exist even within the cortisol values. Finally, the fact that cortisol and fMRI data were collected 1 week apart in the CHR groups could have contributed to the observed lack of association. Future studies could consider fMRI paradigms that incorporate fear and/or stress induction with concomitant cortisol sampling, which would omit the need for the TSST and MRI to be conducted on the same day, which may be burdensome for CHR patients.

We did not observe the expected coupling between TSST-induced anxiety and mediotemporal response to fear processing in healthy individuals, nor any significant group differences in these relationships. While the control vs placebo group interaction was non-significant (*p* = 0.054), CHR patients in the placebo group who deactivated limbic circuitry *less* appeared to have higher TSST-induced anxiety, which would be in keeping with the general consensus discussed above—that mediotemporal deactivation is a neural signature of adaptive regulatory response/resilience to the psychological effects of stress. However, given the lack of significant differences and the relatively small sample sizes available for the anxiety outcome (with only 12 patients with complete data in each CHR group), it is possible that these analyses were underpowered.

Contrary to our exploratory hypotheses, we did not observe a recovery/normalisation of the cortisol-mediotemporal coupling by CBD in CHR patients. CBD attenuates limbic activation in healthy individuals [96, 97] and in patients with anxiety disorders [42], and has anxiolytic effects [43, 98] in people with social anxiety disorder [41, 99] and in healthy people subjected to simulated public speaking [43, 100–102]. In our previous papers in the same patient sample, we demonstrated that 7-day treatment with CBD partially attenuated the neuroendocrine and anxiety response to stress in CHR individuals [40], and a single dose of CBD was sufficient to alter brain activation in CHR individuals during the fear-processing task, in a direction indicative of normalisation [48]. We therefore hypothesised that, if CHR individuals under placebo conditions displayed altered neural-cortisol relationships, that CHR individuals treated with CBD would show at least partial restoration of the normative coupling. It must be noted, however, that indices of brain function and cortisol release were measured under different conditions; the CBD group had their MRI after a single acute dose of CBD (on day 1), while cortisol was measured 7 days later after 600 mg/day dosing. It therefore remains possible that the brain response to fear, if collected also after 7-day CBD treatment, would show the restoration of coupling that we observed in the healthy control group. A further limitation is that the dose of CBD may not have been optimised for anxiolytic effects. While 600 mg, even as a single acute dose, has demonstrable effects on symptoms and brain function across numerous paradigms and clinical populations [46, 48, 61, 103–105], previous work points to an inverted U-shaped response for anxiolytic properties [100, 101, 106]. Finally, with only 12 participants with complete data in the CBD group, those analyses may have been underpowered. For these reasons, our investigation of CBD within this study should be treated as exploratory.

Our results should be considered in the context of certain limitations. First, healthy controls took part in the TSST and MRI scan on the same day, and thus it could be argued that their fMRI data may be contaminated by the residual effects of stress exposure. However, previous work shows that cortisol returns to pre-stressor levels within 41–60 min [65] and scanning was typically acquired about 3 h after the TSST experiment. Second, the cortisol data were not collected during the fMRI scan and the nature of our analyses were correlational. It could, therefore, be argued that our two experimental components (TSST and fear-processing fMRI) were measuring different phenomena—response to social stress vs fear/threat-related stimuli, respectively. Although evidence suggests that fear/threat-related stimuli (i.e., akin to challenges to physical self-preservation) may trigger the HPA axis via activation of the amygdala, while social stress (i.e., challenges to social self-preservation/status) rather involves inhibition of the hippocampus [34, 37], both types of stressor can ultimately converge on the HPA axis [65] and as discussed in the Introduction, fear and HPA axis responses are intertwined on a neuronal as well as neuroendocrine level. In addition, the main effect of our fMRI fear task (exemplified by the task network in healthy controls) was, in fact, parahippocampal deactivation. Nevertheless, future studies employing an overt stress-induction fMRI task (ideally selected so as to also robustly (dis)engage the hippocampal region), along with concomitant cortisol monitoring, would allow further and more direct exploration of these relationships. Future studies could also use longer fMRI paradigms with a greater number of events to further improve signal-to-noise ratio. Another consideration is that we did not evaluate the − 60, + 10 and + 20 min time points, which index the baseline and recovery of the cortisol/anxiety response. The + 0 time point (time B; which occurs 20 min following the onset of the stressor) was selected for our analyses as this represents the hypothetical peak of the cortisol response [65], as well as the peak in anxiety (for all groups) and cortisol (in healthy controls) that we observed in our sample (Supplementary Results) as well as in our related publication [40]. Relatedly, a further cortisol sample (e.g., at − 20 min) would have been advantageous for examining immediate pre-to-post TSST cortisol levels, given that our current baseline measure at − 60 min (time A) may have been affected by “white-coat fear” associated with initial venepuncture, which itself represents a potential stressor. However, these time A measures were not used in the present regression analyses. Nevertheless, we sought to mitigate the overall effects of venepuncture by using an atraumatic needle and an intravenous cannula, which avoided the need for repeated venepuncture. While we cannot rule out the possibility that venous blood sampling may have added to the stress of participants, we believe such stress would have acted across all participants rather than confounding by any particular group. Future studies should follow the protocol outlined by Engert and colleagues [85] and ensure that an adequate number of samples are collected at optimum timepoints, giving consideration to the temporal dynamics of anticipatory vs reactive cortisol release [85] and to the use of alternative cortisol sampling methods, such as salivary testing. Finally, given the exploratory nature of this study, we did not perform multiplicity correction; as such, while we hope that our findings may be useful for future hypothesis generation, our results should be interpreted with caution and replicated in larger samples. Future highly powered studies, particularly those designed to specifically test cortisol responses (in optimum conditions), would also allow for control of potential factors that may influence cortisol response to the TSST, such as other (e.g., over-the-counter) medications, night-shift work [107], body mass index [108], menstrual cycle and the use of contraceptives [109].

Notwithstanding these limitations, our preliminary findings suggest that the parahippocampal (deactivation) response to fear processing may be associated with the neuroendocrine (cortisol) response to experimentally induced social stress, and that this relationship may be altered in patients at clinical high risk for psychosis. Given that environmental stress is a significant but modifiable risk factor for psychosis onset, further work to understand the mechanisms underlying (and potential treatments for) stress intolerance, maladaptive HPA axis responses and increased vulnerability to environmental stress in these individuals is of critical importance.

## Supplementary Information

Below is the link to the electronic supplementary material.

Supplementary file1 (PDF 617 KB)
