# Supplementary material for: Altered relationship between cortisol response to social stress and mediotemporal function during fear processing in people at clinical high risk for psychosis: a preliminary report
Source: Eur Arch Psychiatry Clin Neurosci. 2021 Sep 4;272(3):461–75. doi: 10.1007/s00406-021-01318-z (PMC8938358; doi:10.1007/s00406-021-01318-z)
Supplement: Supplementary file 1 — Supplementary file1 (PDF 617 KB) [file 406_2021_1318_MOESM1_ESM.pdf]

ALTERED RELATIONSHIP BETWEEN CORTISOL RESPONSE TO SOCIAL STRESS AND MEDIOTEMPORAL FUNCTION DURING FEAR PROCESSING IN PEOPLE AT CLINICAL HIGH RISK FOR PSYCHOSIS: A PRELIMINARY REPORT

Cathy Davies, Elizabeth Appiah-Kusi, Robin Wilson, Grace Blest-Hopley, Matthijs G Bossong, Lucia Valmaggia, Michael Brammer, Jesus Perez, Paul Allen, Robin M Murray, Philip McGuire, Sagnik Bhattacharyya

Online Supplementary Material

SUPPLEMENTARY METHODS

Participants

CHR status was determined using the Comprehensive Assessment of At-Risk Mental States (CAARMS) criteria [1]. Briefly, subjects met one or more of the following subgroup criteria: (a) attenuated psychotic symptoms, (b) brief limited intermittent psychotic symptoms (BLIPS, psychotic episode lasting <1 week, remitting without treatment), or (c) either schizotypal personality disorder or first-degree relative with psychosis, all coupled with functional decline [1]. All CHR participants were antipsychotic naive.

Design, Materials, Procedure

The 600 mg dose of CBD was selected based on previous findings that doses of 600-800 mg/day are effective in established psychosis [2] and anxiety [3–5]. The 180 min interval between drug administration and fMRI acquisition was selected based on previous findings describing peak plasma concentrations at 180 min following oral administration [6,7].

Functional Magnetic Resonance Imaging

Image Acquisition

All scans were acquired on a General Electric Signa HDx 3T MR system. Functional images were acquired using Echo Planar Imaging (EPI) with parameters: TR=2000ms, TE=30ms, flip angle=75°, 39×3mm slices, 3.3mm slice gap, matrix= 64×64, FoV=240, 180 timepoints. T1-weighted structural images (inversion recovery EPI; TE=30ms, TR=3000ms, 43×3mm slices, FoV=240mm, matrix=128×128) were also acquired for co-registration.

fMRI Task

Participants were studied in one 6-minute fMRI experiment while performing a fearful face processing task (described in detail elsewhere [8–11]). In short, the blood-oxygen-level-dependent (BOLD) haemodynamic response was measured using an event-related design while subjects viewed fearful faces (mild fear, intense fear), which were contrasted with faces with neutral expressions. Ten different facial identities each conveying a neutral, mild fear and intense fear expression (30 different facial stimuli) were presented twice each for 2s, resulting in 60 facial stimuli in total. The order of presentation of facial identities and expression type was pseudorandomised such that the same identity or expression type was not presented in successive trials. The inter-trial interval was varied from 3–8 seconds according to a Poisson distribution, with an average interval of 4.9 seconds. A fixation cross was presented during the inter-stimulus interval. Participants were asked to indicate the gender of the face via button

press, with the speed and accuracy of responses recorded online throughout image acquisition.

fMRI Analysis

Functional MRI data were analysed with XBAM software version 4.1 using a nonparametric approach to minimise assumptions [12,13]. For each group (control, placebo, CBD), we contrasted the active task condition (mild and intensely fearful faces) against the baseline condition (neutral faces), to identify the brain regions engaged by the processing of fear after controlling for activation related to face processing independent of emotional expression.

Images were corrected for motion [14] and smoothed with a 5mm Gaussian filter. Individual activation maps were created using 2 γ-variate functions to model the BOLD response [15]. Following a least-squares fitting of this model, the sum of squares (SSQ) ratio statistic (ratio of the model component to the residual sum of squares) was estimated at each voxel, followed by permutation testing to determine significantly activated voxels specific to each condition (neutral, mild fear, intense fear) [16,17]. SSQ ratio maps for each individual were transformed into standard stereotactic space [13,18]. Group activation maps for each condition (and then for neutral vs mild fear; and neutral vs intense fear) were computed for each group (control, CBD, placebo) by determining the median SSQ ratio at each voxel (over all individuals). Mild and intense fear were thereafter analysed as a single fearful faces condition.

Group (control, CBD, placebo) activation maps for fearful vs neutral conditions were compared using nonparametric analysis of variance (ANOVA) [12] to examine linear relationships in brain activation (placebo group > CBD group > control group; or placebo group < CBD group < control group). A region-of-interest (ROI) approach was used after constructing a single ROI mask using the Talairach atlas, which included the limbic structures of the bilateral medial temporal lobe (hippocampus, parahippocampal gyrus and amygdala) and the striatum/pallidum (caudate, putamen and globus pallidus). These regions were selected *a priori* based on our previous findings [11,19]. Any results in the striatum/pallidum were not of interest in the present study and were therefore omitted from further/regression analyses. The voxel-wise statistical threshold was set at P=.05, and the cluster-wise thresholds were adjusted to ensure that the number of false-positive clusters per brain would be less than 1; clusters that survived this critical statistical threshold and the corresponding P values are reported. For completeness, corresponding results from wholebrain analyses are presented in Table S2 below.

fMRI Task Performance Analyses

In our previous related paper [11] we assessed task performance (gender discrimination accuracy and response times). While the sample of healthy controls in the current study was partially non-overlapping, we did not have any hypothesis regarding task performance in the present study. We therefore refer readers to our related publication [11].

Trier Social Stress Test (TSST)

This stress induction paradigm involves a social evaluative element, which enhances ecological validity given the comparability with the type of social stressors which individuals may experience in their daily lives. Participants were told they will take part in a public speaking exercise. The experimenter takes the participant into a separate room where a panel of two people were assembled and the standardised TSST instructions were read to them. They were then taken to a different room for the 10 min preparation period. They were informed that they would be given 10 min to prepare for a 5 min speech where they had to imagine they had an interview for their ideal job and they needed to deliver the speech to convince the panellists as to why they were suitable for that job. They were then led to an empty room to prepare. After the 10 min preparation period in a different room, participants returned to the panel to deliver their speech. Once they had completed the speech, they were informed they would take part in a mental arithmetic task as per the TSST protocol (below).

TSST Protocol

Instructions for Experimenter

- Explain to the participant that they will now take part in a social speaking exercise. Go to the room where the committee are assembled.
- "These trained interviewers are here to assess how outgoing, gregarious, and comfortable you are in situations in which you must project yourself as an expert. This is a type of personality test for a trait called extraversion. We are asking you to imagine that you are applying for your ideal job. You have dreamed about working in this job for as many years as you can remember. You have just seen an advertisement for this perfect job and decided to apply. After submitting your application, you have been invited for an interview. The job pays a very large salary. You are competing against a lot of other candidates, and the final selection will be made based on your ability to convince the interviewers of how your experiences, abilities, and education make you a better candidate that the others. You will try to convince this panel of interviewers that you are the best candidate for the position. In addition, you will be asked to perform a mental math test, which will give us additional information about your working memory capacity. You will have 10 minutes to prepare a detailed speech lasting 5 minutes. After the preparation time has elapsed, you will return and deliver your speech to these interviewers. Your speech should explain why you should get the job. Remember, you should try to perform better than all of the other participants. These examiners are specially trained to monitor and rate your speech for its believability and convincingness, and they will compare your performance to that of the others who perform this task. Also, you will be videotaped during the task so that the examiners can go over the videotape carefully and rate the contents of your speech as well as your nonverbal behaviour. Now let us go back to your room so that you can prepare for your job interview in the given 5 minutes."
- Take participant to preparation room and ask them to prepare (the room should have lined paper and pens and pencils). Answer any questions.
- Leave the Room.
- Come back 10 minutes later ask if they have any questions and then take the participant to the interview room.
- "I will now hand you over to the committee, your participant number is….."
- Stand in corner of the room, do not make eye contact with the participant, and allow committee members to be in charge.
- Once the test is over, take the participant to the clinical room to collect blood samples.

Instructions for Committee

The committee has contact twice with the participant during the introduction and during the actual stress phase. One member should assume the role of chairperson.

Introduction

During the introduction the only duty of the committee is to be present. You should avoid talking to the participant during the introduction phase.

Part 1

The actual task of the committee starts when the participant enters the room ten minutes after the introduction to deliver his/her speech.

- Turn on the video camera
- Seek eye contact with the participant during the speech
- Let the participant speak for the first three minutes.
- If the participant stops before then, there should be a pause. After about a twenty seconds pause the chairperson can alert the participant to the remaining time by saying: “You still have time, please continue”
- If after another ten seconds the participant doesn’t have more to say, you should ask questions for the remaining time. For example:
    - Why do you think you are the best applicant?
    - What other experiences do you have in this area?
- In rare instances will the participant be able to talk alone for the full five minutes. In that case allow the participant to continue but stop them at 5 minutes

Part 2

- After, the 5 minutes, tell the participant: “That will be enough for now”
- “We now want to ask you to work on a second task. This one is about mental arithmetic. We ask you to count backwards to zero in 13-number steps, starting at 1022, and to do it as fast and correctly as possible. Should you miscalculate, you will be told so and you start again at 1022. Do you have any questions about this?...Please begin, then."
- Should the subject miscalculate, the chair will respond with the standard phrase "Error. 1022." until the end of the test period.
- At the end of the test period the chair should thank the participant for their participation. They will be led out by the experimenter.

SUPPLEMENTARY RESULTS

Region of Interest (ROI) fMRI Results

Table S1. Linear Relationship in Activation Across 15 Participants at Clinical High Risk for Psychosis receiving Placebo (PLB), 16 Healthy Controls (HC), and 14 CHR Participants Receiving Cannabidiol (CBD) - ROI

Region	Talairach Coordinates x	y	z	Cluster Size, No. of Voxels	P value[a]
Placebo > CBD > Controls					
Parahippocampal Gyrus	-22	-26	-17	6	<.001
Parahippocampal Gyrus	25	-48	-3	26	<.001
Fusiform Gyrus	-25	-56	-7	10	<.001
Controls > CBD > Placebo					
Putamen	-18	7	0	12	<.001
Caudate Head	-7	19	0	3	<.001

[a] Corrected for less than 1 false-positive cluster.

Supplemental Wholebrain fMRI Results

Table S2. Linear Relationship in Activation Across 15 Participants at Clinical High Risk for Psychosis receiving Placebo (PLB), 16 Healthy Controls (HC), and 14 CHR Participants Receiving Cannabidiol (CBD) - Wholebrain

Region	Talairach Coordinates x	y	z	Cluster Size, No. of Voxels	P value[a]
Placebo > CBD > Controls					
Anterior lobe of Cerebellum extending to Fusiform Gyrus	-25	-41	-26	42	.002
Cerebellum (Declive) extending to Parahippocampal Gyrus	-33	-56	-7	186	<.001
Parahippocampal Gyrus extending to Culmen & Lingual Gyrus	25	-48	-3	74	<.001
Controls > CBD > Placebo					
Fusiform Gyrus	40	-59	-10	22	.003
Caudate Head (bilateral) extending to Putamen, Anterior Cingulate, Medial & Middle Frontal Gyri	-11	22	0	183	<.001
Postcentral Gyrus extending to Inferior Parietal Lobule	-40	-22	53	104	<.001

[a] Corrected for less than 1 false-positive cluster.

Figure S1. Clusters from wholebrain analyses where activation differed across the 3 groups in a linear relationship during fear processing. Regions where Controls > CBD > Placebo are shown in red; regions where Placebo > CBD > Controls are shown in blue. The right side of the brain is shown on the left of the images. Please note that the Talairach-derived clusters below are overlaid on a standard (MNI) brain template for display purposes only.

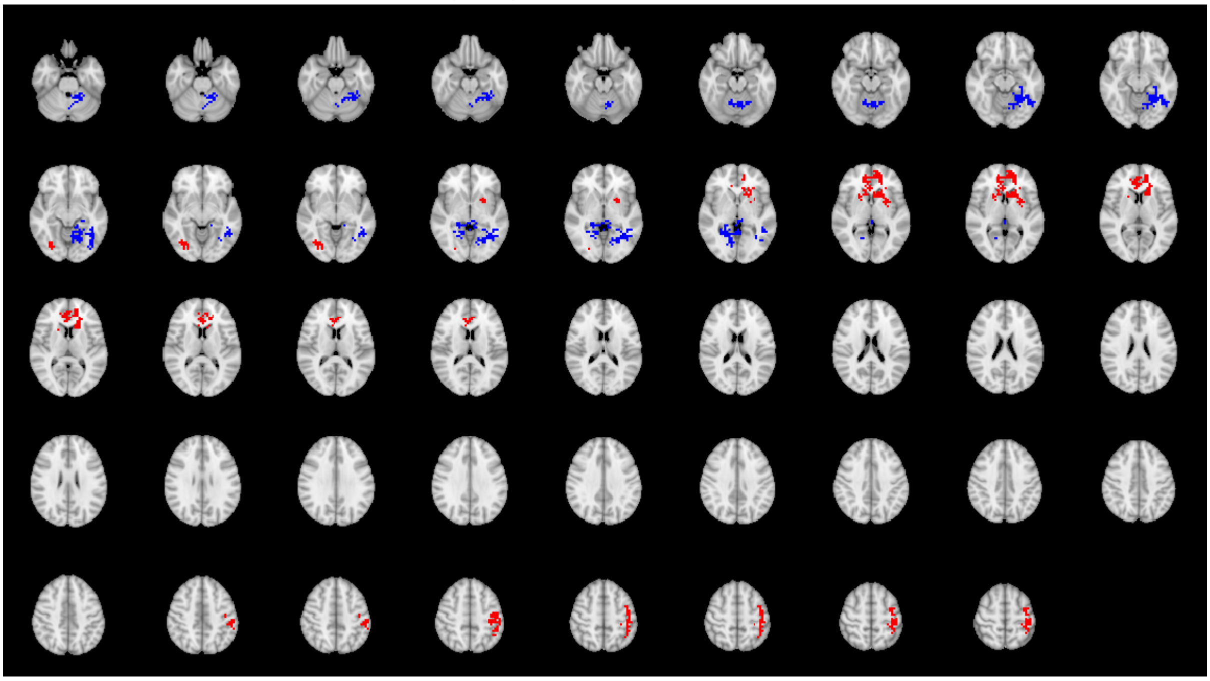

Cortisol response to acute stress induction with TSST

Figure S2. Mean cortisol levels over the course of the TSST in the healthy control (HC), placebo (PLB) and cannabidiol (CBD) groups. The data depicted below have been described (in a mostly overlapping sample) in our previous paper [20].

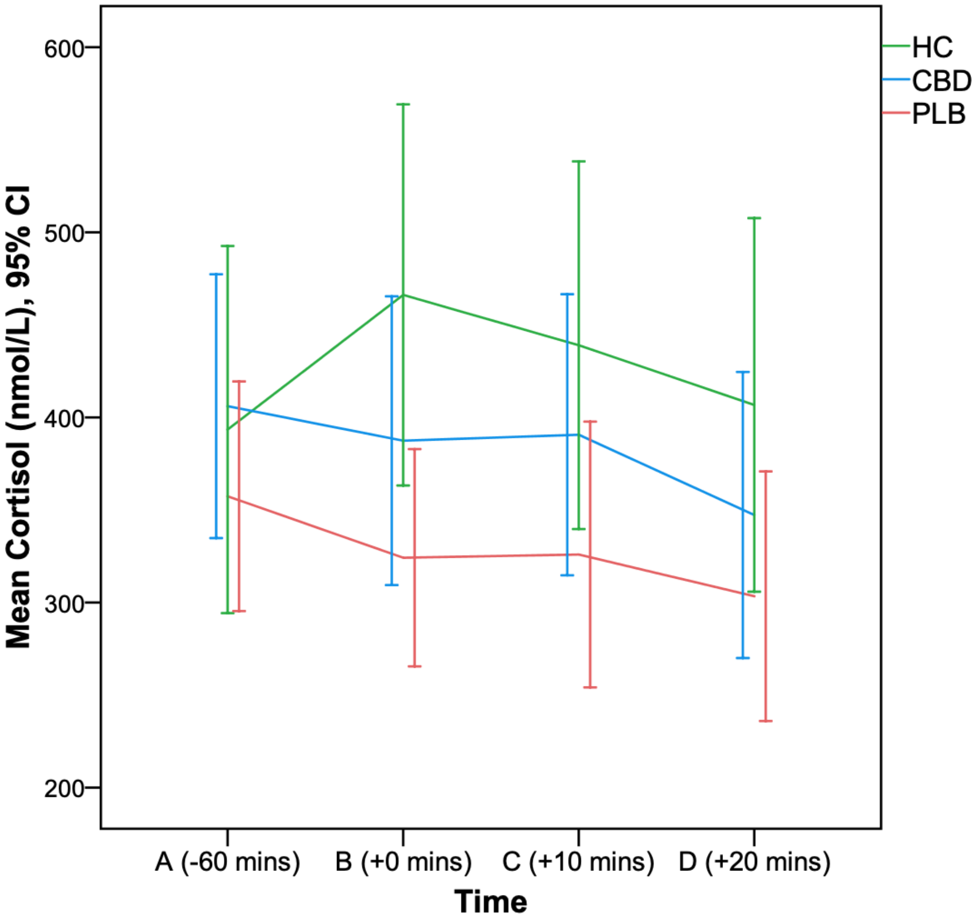

Table S3. Mean cortisol levels (nmol/L) over the course of the TSST in the healthy control (HC), placebo (PLB) and cannabidiol (CBD) groups.

	HC (total N=16)			CBD (total N=14)			PLB (total N=15)		
Timepoint	Mean	SD	N data*	Mean	SD	N data*	Mean	SD	N data*
A (-60 mins)	393.44	186.13	16	406.07	123.44	12	357.40	112.02	13
B (+0 mins)	466.19	193.22	16	387.43	135.11	11	324.20	105.98	13
C (+10 mins)	439.00	186.39	16	390.64	131.49	12	325.93	129.63	13
D (+20 mins)	406.75	189.39	16	347.29	133.86	13	303.40	121.75	13

*For cortisol analyses, if a subject's data were missing for a specific timepoint they were imputed using last observation carried forward (LOCF). This table shows the descriptive summary results after this imputation. Therefore, the difference between the total group N and the value in the "N data" column (which shows the number of subjects with data) is the number of subjects for whom cortisol values were imputed for that timepoint.

Supplementary Statistical Analyses – Cortisol

Given that the sample was not identical to the sample in our previous paper, we conducted supplementary statistical analyses to test whether the TSST procedure evoked a significant cortisol stress response. In line with our previous paper, SPSS (version 27.0) was used for these additional analyses.

Effect in Healthy Controls

Using a paired t-test to compare cortisol levels at time A vs time B, we confirmed that the TSST procedure evoked a significant stress response in healthy controls: t(15) = -2.38, p=.031 [two-tailed]; time A vs time B mean difference= -72.75, 95% CI -137.57 to -7.93; Fig S2, Table S3).

Group differences in change in cortisol

To confirm concordance with our previous related paper [20], we conducted a supplementary one-way ANOVA to compare the change in cortisol from time A to time B (B minus A) between all 3 groups, followed by pairwise contrasts (independent t-tests) using this change score. We found a significant effect of group (HC, PLB, CBD) on change in cortisol levels F(2,42)= 3.33, p=.045). Pairwise contrasts showed that the change in cortisol levels was significantly different (t(29)= 2.22, p=.034) in HC (change M=72.75, SD= 121.64, n=16) compared with PLB (M= -33.20, SD= 143.90, n=15), and in HC when compared with CBD (M= -18.64, SD= 101.60, n=14; t(28)= 2.21, p=.035), but was not different between PLB and CBD (t(27)= -.313, p= .76).

Anxiety response to acute stress induction with TSST

Figure S3. Mean anxiety scores over the course of the TSST in the healthy control (HC), placebo (PLB) and cannabidiol (CBD) groups. The data depicted below have been described (in a mostly overlapping sample) in our previous paper [20].

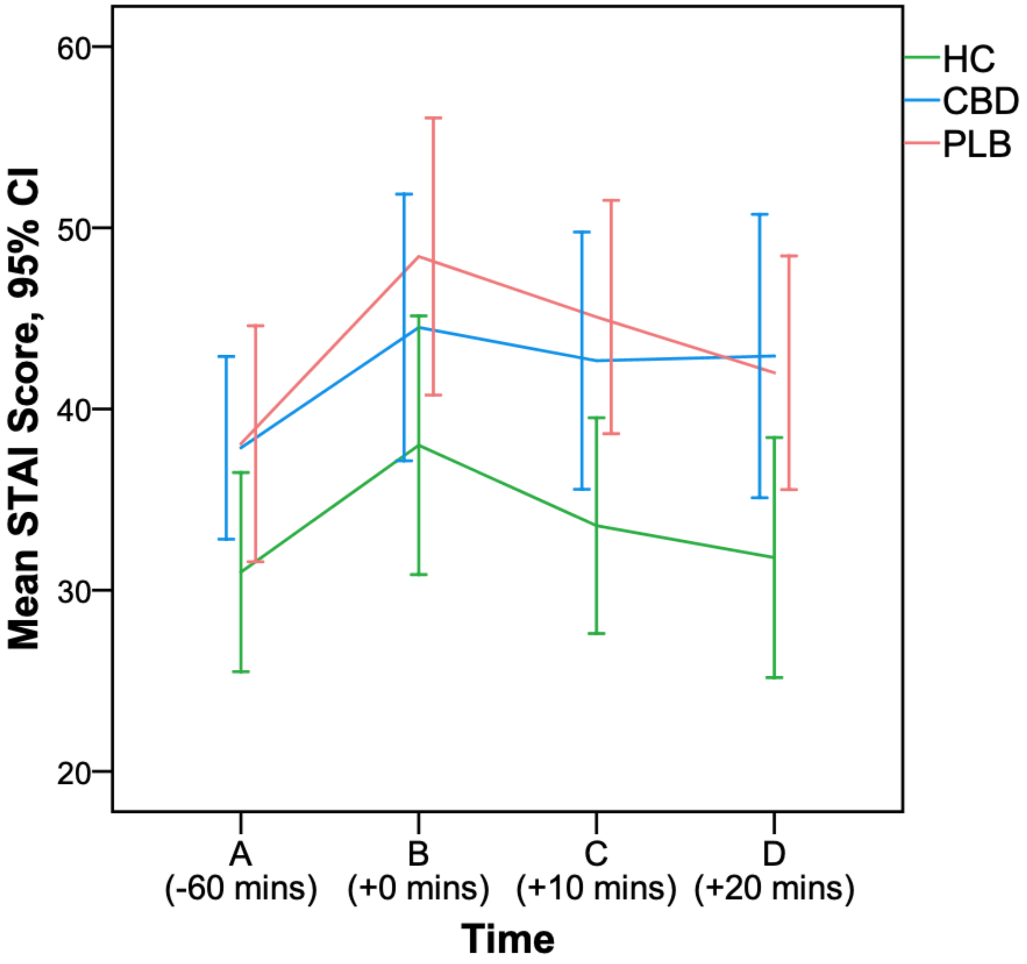

Table S4. Mean STAI scores over the course of the TSST in the healthy control (HC), placebo (PLB) and cannabidiol (CBD) groups.

	HC (total N=16)			CBD (total N=14)			PLB (total N=15)		
Timepoint	Mean	SD	N data*	Mean	SD	N data*	Mean	SD	N data*
A (-60 mins)	31.00	9.52	14	37.86	8.73	14	38.08	10.25	12
B (+0 mins)	38.00	13.40	16	44.50	11.58	12	48.42	12.03	12
C (+10 mins)	33.56	11.17	16	42.67	11.17	12	45.08	10.66	13
D (+20 mins)	31.80	11.96	15	42.92	12.95	13	42.00	10.67	13

*For STAI analyses, if a subject's data were missing for a specific timepoint they were not imputed and simply removed from the analyses/dataset. This table therefore shows the descriptive summary results using all available data and without imputation. Therefore, the value in the "N data" column (which shows the number of subjects with data) is the number of subjects for whom these descriptive summary statistics are based on.

Supplementary Statistical Analyses – Anxiety

Given that the sample was not identical to the sample in our previous paper, we conducted supplementary statistical analyses to test whether the TSST procedure evoked a significant anxiety response. In line with our previous paper, SPSS (version 27.0) was used for these additional analyses.

Effect in Healthy Controls

Using a paired t-test to compare STAI scores at time A vs time B, we confirmed that the TSST procedure evoked a significant anxiety response in healthy controls: $t(13) = -2.42$, $p=.031$ [two-tailed]; time A vs time B mean difference= -7.71, 95% CI -14.60 to -0.83; Fig. S3, Table S4).

Group differences in change in STAI scores pre-to-post TSST

We conducted a supplementary one-way ANOVA to compare the change in STAI scores from time A to time B (B minus A) between all 3 groups. There was no significant difference between groups ($F(2,34)=.107$, $p=.90$).

Area Under the Curve Analyses

Area under the curve (AUC) for the four time points was calculated using the trapezoid formula with respect to ground, as outlined by Pruessner et al. [21], and used in a one-way ANOVA with follow-up pairwise contrasts (independent t-tests) to test concordance with our previous related paper [20]. Akin to the previous study, we found a significant effect of group (HC, PLB, CBD) on STAI AUC scores $F(2,33)= 3.89$, $p= .030$). Pairwise contrasts showed that the STAI AUC was significantly different ($t(22)= -2.60$, $p=.016$) in HC (M=1353.46, SD= 414.59, n=13) compared with PLB (M=1795.91, SD= 415.94, n=11), but not in HC when compared with CBD (M=1663.75, SD= 374.40, n=12; $t(23)= -1.96$, $p=.062$), and was also not different between PLB and CBD ($t(21)= .80$, $p=.43$).

REFERENCES

1. Yung AR, Pan Yuen H, McGorry PD, Phillips LJ, Kelly D, Dell'olio M, et al. Mapping the Onset of Psychosis: The Comprehensive Assessment of At-Risk Mental States. Aust New Zeal J Psychiatry. 2005;39:964–971.
2. Leweke FM, Piomelli D, Pahlisch F, Muhl D, Gerth CW, Hoyer C, et al. Cannabidiol enhances anandamide signaling and alleviates psychotic symptoms of schizophrenia. Transl Psychiatry. 2012;2:e94–e94.
3. Crippa JA, Guimarães FS, Campos AC, Zuardi AW. Translational Investigation of the Therapeutic Potential of Cannabidiol (CBD): Toward a New Age. Front Immunol. 2018;9:1–16.
4. Lee JLC, Bertoglio LJ, Guimarães FS, Stevenson CW. Cannabidiol regulation of emotion and emotional memory processing: relevance for treating anxiety-related and substance abuse disorders. Br J Pharmacol. 2017;174:3242–3256.
5. Bergamaschi MM, Queiroz RHC, Chagas MHN, De Oliveira DCG, De Martinis BS, Kapczinski F, et al. Cannabidiol reduces the anxiety induced by simulated public speaking in treatment-nave social phobia patients. Neuropsychopharmacology. 2011;36:1219–1226.
6. Millar SA, Stone NL, Yates AS, O'Sullivan SE. A systematic review on the pharmacokinetics of cannabidiol in humans. Front Pharmacol. 2018;9.
7. Martin-Santos R, A. Crippa J, Batalla A, Bhattacharyya S, Atakan Z, Borgwardt S, et al. Acute Effects of a Single, Oral dose of d9-tetrahydrocannabinol (THC) and Cannabidiol (CBD) Administration in Healthy Volunteers. Curr Pharm Des. 2012;18:4966–4979.
8. Fusar-Poli P, Crippa JA, Bhattacharyya S, Borgwardt SJ, Allen P, Martin-Santos R, et al. Distinct Effects of Δ9-Tetrahydrocannabinol and Cannabidiol on Neural Activation During Emotional Processing. Arch Gen Psychiatry. 2009;66:95.
9. Bhattacharyya S, Morrison PD, Fusar-Poli P, Martin-Santos R, Borgwardt S, Winton-Brown T, et al. Opposite effects of delta-9-tetrahydrocannabinol and cannabidiol on human brain function and psychopathology. Neuropsychopharmacology. 2010;35:764–774.
10. Bhattacharyya S, Egerton A, Kim E, Rosso L, Riano Barros D, Hammers A, et al. Acute induction of anxiety in humans by delta-9-tetrahydrocannabinol related to amygdalar cannabinoid-1 (CB1) receptors. Sci Rep. 2017;7:15025.
11. Davies C, Wilson R, Appiah-Kusi E, Blest-Hopley G, Brammer M, Perez J, et al. A single dose of cannabidiol modulates medial temporal and striatal function during fear processing in people at clinical high risk for psychosis. Transl Psychiatry.

2020;10:311.
12. Brammer MJ, Bullmore ET, Simmons A, Williams SCR, Grasby PM, Howard RJ, et al. Generic brain activation mapping in functional magnetic resonance imaging: A nonparametric approach. Magn Reson Imaging. 1997;15:763–770.
13. Thirion B, Pinel P, Mériaux S, Roche A, Dehaene S, Poline JB. Analysis of a large fMRI cohort: Statistical and methodological issues for group analyses. Neuroimage. 2007;35:105–120.
14. Bullmore ET, Brammer MJ, Rabe-Hesketh S, Curtis V a, Morris RG, Williams SC, et al. Methods for diagnosis and treatment of stimulus-correlated motion in generic brain activation studies using fMRI. Hum Brain Mapp. 1999;7:38–48.
15. Friman O, Borga M, Lundberg P, Knutsson H. Adaptive analysis of fMRI data. Neuroimage. 2003;19:837–845.
16. Bullmore E, Long C, Suckling J, Fadili J, Calvert G, Zelaya F, et al. Colored noise and computational inference in fMRI time series analysis : resampling methods in time and wavelet domains SInstitute of Psychiatry KCL , London UK. Hum Brain Mapp. 2001;78:2001.
17. Bullmore ET, Suckling J, Overmeyer S, Rabe-Hesketh S, Taylor E, Brammer MJ. Global, voxel, and cluster tests, by theory and permutation, for a difference between two groups of structural MR images of the brain. IEEE Trans Med Imaging. 1999;18:32–42.
18. Talairach J, Tournoux P. Co-Planar Stereotaxis Atlas of the Human Brain: an approach to cerebral imaging. New York: Thieme Medical Publishers; 1988.
19. Bhattacharyya S, Wilson R, Appiah-Kusi E, O’Neill A, Brammer M, Perez J, et al. Effect of Cannabidiol on Medial Temporal, Midbrain, and Striatal Dysfunction in People at Clinical High Risk of Psychosis: A Randomized Clinical Trial. JAMA Psychiatry. 2018;75:1107–1117.
20. Appiah-Kusi E, Petros N, Wilson R, Colizzi M, Bossong MG, Valmaggia L, et al. Effects of short-term cannabidiol treatment on response to social stress in subjects at clinical high risk of developing psychosis. Psychopharmacology (Berl). 2020;237:1121–1130.
21. Pruessner JC, Kirschbaum C, Meinlschmid G, Hellhammer DH. Two formulas for computation of the area under the curve represent measures of total hormone concentration versus time-dependent change. Psychoneuroendocrinology. 2003;28:916–931.
